# An Algorithm to Analyze Cost Heterogeneity using Counterfactual Scenarios in Endovascular versus Open Repair of Abdominal Aortic Aneurysm: Predicting Costs for Subsequent Patients

**Published:** 2014-02-26

**Authors:** Christopher A. Jones, Peter W. Callas, Robert W. Everett, Richard A. Galbraith, Richie Spitsberg, Jeffrey J. Petrozzino, Michael J. DeSarno, Andrew C. Stanley

**Affiliations:** 1 Global Health Economics Unit of the Vermont Center for Clinical and Translational Science, University of Vermont, Burlington, VT, USA; Department of Surgery, University of Vermont College of Medicine, Burlington, VT, USA; Department of Economics, University of Vermont, Burlington, VT, USA; European Centre for International Political Economy, Brussels Belgium; 2 Department of Biostatistics University of Vermont College of Medicine, Burlington, VT, USA; 3 Global Health Economics Unit of the Vermont Center for Clinical and Translational Science University of Vermont, Burlington, VT, USA; 4 Global Health Economics Unit of the Vermont Center for Clinical and Translational Science, University of Vermont, Burlington, VT, USA; Vermont Center for Clinical and Translational Science, University of Vermont, Burlington, VT, USA; 5 Department of Surgery, University of Vermont College of Medicine, Burlington, VT, USA; Fletcher Allen Health Care, Burlington, VT, USA

**Keywords:** health economics, aneurysm repair, vascular surgery, forward simulation, point-of-care cost analysis, predictive model

## Abstract

**Objectives:** We examined patient-specific predictors of high cost for endovascular (EVAR) and open (OPEN) repair of abdominal aortic aneurysm (AAA).

**Methods:** Vascular Study Group of Northern New England data specific to Fletcher Allen Health Care were merged with cost data from the same source. We retrospectively analyzed 389 elective AAA repairs (230 EVAR, 159 OPEN) between 2003 and 2011 to determine clinical characteristics that contribute to membership in the upper quartile of cost (UQC) versus the remaining three quartiles. For the purpose of this exercise, it was assumed that clinical outcomes were equally good with EVAR versus OPEN repair.

**Results:** Significant predictors of UQC for OPEN repair procedures were: history of treated chronic obstructive pulmonary disease (COPD), previous bypass surgery, transfer from hospital and age >70 (area under receiver operating curve [ROC] = 0.726). Predictors of UQC for EVAR were: presence of iliac aneurysm(s), coronary artery bypass graft surgery or percutaneous transluminal coronary angioplasty within the past 5 years, ejection fraction ≤30%, absence of beta blockers, creatinine ≥1.5mg/dL, and current use of tobacco (area under ROC = 0.784). The mean length of stay for EVAR and OPEN repair were 2.22 and 8.55 days, respectively. Costs for EVAR and OPEN repair were $32,656 (standard error of the mean [SEM] $591) and $28,183 (SEM $1,571), respectively.

**Conclusions:** Certain risk factors at the individual patient level are predictive of UQC. Under such circumstances, it is our expectation that such algorithms may be used to select the most cost-efficient treatment.

[1]Global Health Economics Unit of the Vermont Center for Clinical and Translational Science, University of Vermont, Burlington, VT, USA; [2]Department of Biostatistics, University of Vermont College of Medicine, Burlington, VT, USA; [3]Department of Surgery, University of Vermont College of Medicine, Burlington, VT, USA; [4]Department of Economics, University of Vermont, Burlington, VT, USA; [5]European Centre for International Political Economy, Brussels Belgium; [6]Fletcher Allen Health Care, Burlington, VT, USA; [7]Vermont Center for Clinical and Translational Science, University of Vermont, Burlington, VT, USA *Corresponding author chris.jones@vtmednet.org

## 1. Introduction

"At a time of finite resources, the benefit provided by new interventions must be weighed against their expense," wrote Chaikof and colleagues and presciently so.[1] Over the last 5 years, the rising costs of healthcare interventions and technologies have become a harbinger for massive country-wide efforts to contain costs. While successful clinical outcomes remain the most important consideration for patients undergoing surgery for abdominal aortic aneurysm (AAA) repair, health economic analyses are increasingly being used to influence healthcare reimbursement decisions in the United States and elsewhere. "Point-of-Care, Cost-prediction Analysis" (POCCA) is a new phraseology which we are introducing to describe a method of predicting the cost, effectiveness and cost-effectiveness (as appropriate) of alternative treatments or procedures for new patients, based on matching the clinical and economic characteristics of these new patients to large numbers of those already evaluated. There is precedent for such a forward-thinking approach. A procedure's cost is increasingly being weighed relative to mandated state and federal funding limits, and to new accountable care metrics whereby hospital organizations are paid as a function of improvements in quality and reductions in readmissions. One example of a medical and technological development that shows clear clinical and possibly financial advantages for certain subsets of patients is the endovascular repair (EVAR) of non-ruptured abdominal aortic aneurysm.

We undertook a POCCA of a single vascular surgery clinic's multi-year data to test whether modeling can show for whom and in what specific circumstances an EVAR versus open (OPEN) repair might lead to higher cost, given the option of undergoing either approach.

## 2. Methods

Our analysis was performed in three sequential steps: 1) We built a model around associations between perioperative variables after demonstrating that clinical heterogeneity existed between patients who underwent EVAR versus OPEN repair during the period of study. 2) We tested for statistical significance in these variables predicting the upper quarticle of cost (UQC); and 3) we examined whether the decision to undergo EVAR versus OPEN repair, based on costs that included any perioperative complications to the point of hospital discharge, would be dominated by one of these options. If the decision was non-obvious, money could be saved by selecting the less expensive treatment personalized to the individual circumstances of the patient.

The dataset used for this analysis of AAA repairs started with 5,199 patients (3,012 EVAR, 2,187 OPEN repair) as compiled prospectively by the Vascular Surgery Group of New England (VSGNE). Detailed descriptions of this dataset can be found in recent publications by Crononwett, *et al.*[2] and Beck, *et al.*[3] We analyzed a subset of this dataset, and focused exclusively on elective AAA patients who received OPEN repair or EVAR in Vermont at the Fletcher Allen Healthcare (FAHC) facility, a total of 389 AAA patients. The dataset is unique in that it prospectively collects information on preoperative, intraoperative and postoperative elements of patient care.

In general, younger healthy patients with unfavorable anatomy are good candidates for OPEN repair, while older patients with significant comorbidities and favorable anatomy have better results when treated with the less invasive EVAR. A significant number of patients presenting with AAA could therefore be treated safely utilizing either modality. The FAHC dataset comprised 389 patients in total, 230 of whom underwent OPEN repair and 159 EVAR. Confidentiality was maintained for all patients included in this study in accordance with institutional standards. Data was collected by the VSGNE over an 8-year period from

January 1, 2003 through December 31, 2011. All costs were included to discharge, and any perioperative complications prior to discharge were included in the total cost. More specifically, along with chart data, corresponding direct and indirect hospital costs along with sum total hospital costs (in 2011 USD) were made available for each patient in a manner that was compliant with institutional review board approval.

Cost data were derived from the general ledger for expenses along with the hospital and professional billing systems that provide service activity volumes for every patient encounter. Two major costs were included in our final analysis: direct and indirect costs.

Direct costs were defined as those associated with any expense booked directly for a specific case. Such costs included ten separate components. The first nine of these were groupings of general ledger accounts that capture the expenses of each episode of patient care. These were: physician labor (personnel time for physicians), non-physician labor (nurses and other staff), clinical supplies, pharmaceuticals, equipment leases, equipment maintenance, purchased services, non-clinical supplies and miscellaneous expenses. The tenth cost component was used to spread the overhead costs that had been allocated to each department across all service activities billed out of that department (vascular surgery, in this context). Given the complexity of purchasing decisions, direct costs for equipment and personnel time were calculated by working backwards from hospital scheduling and based upon a full-absorption revenue value unit (RVU) model. All costs for the organization were allocated to service activity codes based on the facility charge master and the medical group professional fee schedules billed by organizational departments.

Indirect costs were defined as departmental expenses that were not specifically associated with a case, but which were allocated as overhead to the department(s) directly involved in the case. They ranged from the costs of specific administrative services to depreciation of equipment and management of buildings, to a share of graduate medical education costs, all assigned through a reciprocal allocation method. The total cost was simply a summation of these direct and indirect costs.

FAHC defined length of stay (LOS) as the total number of overnights a patient remained in the hospital, and an initial analysis was conducted to compare direct and indirect costs with the patient's LOS.

Analyses were conducted separately for OPEN and EVAR AAA patients. Descriptive statistics were first computed to detect anomalous values and/or outliers. Categories were combined for some variables due to small numbers of patients in certain of the categories. For elective open AAA patients, for example, only one patient had transferred from a rehabilitation unit, so they were combined with the field "Transferred from a Hospital" to create a combined category of "Transferred from Hospital or Rehabilitation Unit."

Bivariate analyses were conducted to compare demographic and preoperative characteristics of patients predicted to be members of the UQC, to patients in the remaining quartiles. Patients were compared on the following variables using chi square tests for categorical variables and two sample t-tests for continuous variables:

- Gender;
- Race;
- Age;
- Body mass index (BMI);
- Transfer status (not transferred/hospital or rehabilitation unit);

- Cigarette smoking (never/prior/current);
- Hypertension (no/yes);
- Diabetes (no/yes);
- Beta-blocker use (no or intolerant/pre-operation or day of operation/chronic);
- Coronary artery disease (CAD) (none/history of myocardial infarction (MI) no symptoms/angina or MI previous 6 months);
- Coronary artery bypass graft (CABG) surgery/percutaneous transluminal coronary angioplasty (PTCA) (none/less than 5 years ago/5 or more years ago);
- Congestive heart failure (no/yes);
- Chronic obstructive pulmonary disease (COPD)(no/not treated/on medication/on home oxygen);
- Dialysis (no/yes or transplant);
- Creatinine (mg/dl);
- Stress test (not done/normal/positive for ischemia/positive for MI);
- Pre-admission living (home/nursing home);
- History of bypass (no/yes);
- History of carotid endarterectomy (no/yes);
- History of aneurysm repair (no/yes);
- History of percutaneous transluminal angioplasty (PTA)/stent (no/yes);
- Aspirin (no or intolerant/yes);
- Plavix (no/yes);
- Statin (no or intolerant/yes);
- Family history of AAA (no/yes);
- Prior aortic surgery (no/yes);
- Ejection fraction (<30%/30-50%/>50% /not done);
- Maximum anteroposterior (AP) AAA diameter (mm); and
- Iliac aneurysm (no/unilateral/bilateral).

A logistic regression was used to predict being in the UQC, with the area under the receiver operating curve (ROC) used to measure the discriminative ability of the model. Variables that significantly (p-value<0.05) or marginally (0.05<p-value<0.10) differed between the upper quartile and other patients on bivariate analysis were initially included in the model. Variables were then removed, one at a time, starting with the least significant, until only independently significant predictors remained in the model. The adequacy of fit of the logistic model to the data was tested using the Hosmer-Lemeshow goodness of fit test.[4] Internal validation of the final model was conducted by bootstrapping, as recommended by Steyerberg, *et al.*,[5] using 1000 bootstrap samples.

Forward simulation algorithms were then created by adopting a regression approach that could be back-tested on the dataset and cross-tested between treatment arms, thus yielding the degree of confidence to which patients who received EVAR would have otherwise accrued less cost if treated with OPEN repair, and vice versa. Our goal was to estimate a separate model for both EVAR and OPEN that could be used to predict total cost as ex ante data are collected on each patient. Weights were estimated using least squares multiple regression. Risk factors that hypothetically could impact total cost for EVAR and OPEN procedures were tested on subsamples of patients. Risk factors were removed until statistically significant factors remained for estimating total cost for both EVAR patients and OPEN patients.

Once the predictive models were developed, they were used to predict cost for both EVAR and OPEN procedures on each patient in both subsets of data. Stated another way, the models were used to test whether EVAR would remain the most cost-efficient option if rather, hypothetically, those same patients were to receive an OPEN repair, and vice versa. Evidence of discriminatory ability between predictive models was then compiled and examined.

In the final comparative analysis, total mean (with standard error of the mean [SEM]) hospital costs for EVAR (International Classification of Disease, $9^{th}$ Revision [ICD-9] code 39.71) versus those for OPEN (ICD-9 code 38.44) procedures were compared between Vermont and a national sampling of countrywide estimates. These national costs were based on the most recent Healthcare Cost and Utilization Project (HCUP) data available for 2010 (http://hcupnet.ahrq.gov), and inflated annually by 5.6% to estimate 2011 costs. The annual 5.6% annual inflation index was based on U.S. Bureau of Labor Statistics consumer price index and a U.S. city average for hospital and related services (www.bls.gov/cpi/cpid11av.pdf). Two-tailed unpaired t-tests were performed based on mean, SEM, and sample size using GraphPad (www.graphpad.com/quickcalcs/ttest1.cfm). Based on the prior finding that all studies to date (based on data between 1995 and 2004) had found that EVAR was more expensive than OPEN AAA surgery,[6] additional national cost data were obtained from 2006 (the "oldest" costing data available from HCUP for EVAR and OPEN procedures) through 2009.

## 3. Results

All patients who underwent EVAR or OPEN repair survived until discharge. A comparison of the preoperative characteristics of the EVAR and OPEN repair patients showed that patients in these groups differed significantly ($p<0.05$) on age, gender, beta blocker use, CABG/PTCA history, congestive heart failure, stress test results, aspirin use, prior aortic surgery, ejection fraction, maximum AP AAA diameter and iliac aneurysm. They differed marginally ($0.05< p<0.10$) on BMI, cigarette smoking, CAD symptoms, COPD, history of bypass and history of PTA/stent.

OPEN repair patients were found to have a LOS ranging from 3-68 days (mean = 8.55; SEM = 0.42), while EVAR patients ranged 1-11 days (mean = 2.22; SEM = 0.145). These findings are consistent with those Matsumura, *et al.*,[7] where the EVAR patients are expected to leave the hospital sooner than OPEN repair patients, as shown in Figure 1. However, this significant difference ($p<0.001$) in LOS did not extrapolate to proportionate differences in total cost. EVAR patients were more expensive than OPEN repair patients, on the average ($p<0.05$) and total costs for either procedure showed a significantly greater than expected variance, ranging from $11,926 to $59,462 (mean = $32,656, SEM = $591) for EVAR and $12,557 to $266,615 (mean = $28,183, SEM = $1,571) for OPEN repair.

Risk factors were regressed relative to total costs in the OPEN repair subsample (n=230). Results are summarized in Table 1. Associated with high cost patients were four preoperative risk factors and comorbidities that have not previously been reported together. These significant ($p<0.05$) variables, which were associated with variation in total cost, were age, transfer status and history of prior bypass and COPD. Based on a locally weighted scatterplot smoother (LOWESS), analysis of continuous age versus the logit of being in the UQC, age dichotomized at 70 years was determined to be the most appropriate definition for the cost prediction modeling. With age not linearly related to the logit of the outcome, this parameter was dichotomized as <70 versus ≥70 years.

**Figure 1. Total Costs for EVAR and Open AAA Repair Patients Based on Length of Stay**

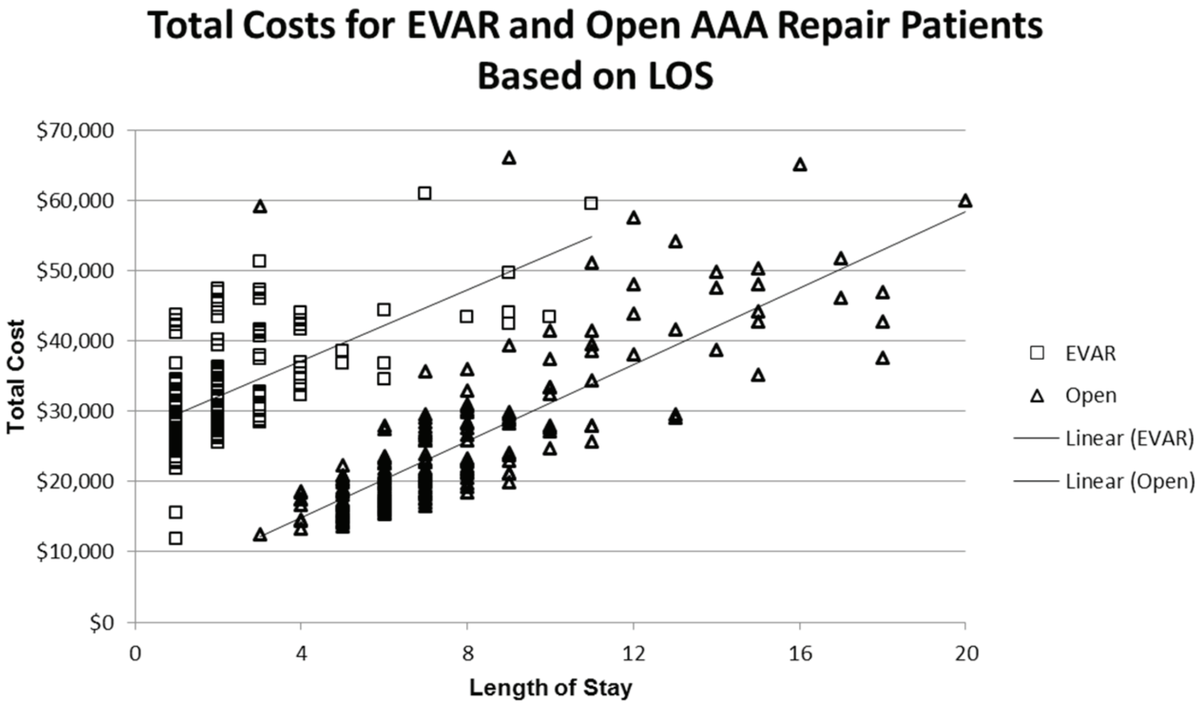

**EVAR Cost**
Mean = $32,656 (SEM = $591)

**OPEN Repair Cost**
Mean = $28,183 (SEM = $1,571)

**EVAR LOS**
Mean = 2.22 (SEM = 0.145)

**OPEN LOS**
Mean = 8.55 (SEM = 0.42)

AAA: abdominal aortic aneurysm; LOS: length of stay; SEM: standard error of the mean; EVAR: endovascular repair

**Table 1. Predictors of Upper Quartile Costs for OPEN Repair Patients**

| Variable | Comparison | OR | 95% CI Lower | 95% CI Upper | P-value |
|---|---|---|---|---|---|
| Age | >70 vs. ≤70 | 3.23 | 1.63 | 6.39 | <0.001 |
| COPD | Yes vs. No | 1.63 | 1.08 | 2.46 | 0.020 |
| Hx Bypass | Yes vs. No | 5.64 | 1.45 | 21.90 | 0.010 |
| Transfer | Yes vs. No | 8.08 | 1.60 | 40.72 | 0.010 |

OR: odds ratio; CI: confidence interval; COPD: chronic obstructive pulmonary disease; Hx: history

The assumption that this logistic model fit the data was satisfied (p=0.61). For the test of model fit, a non-significant p-value indicates fit. Stated another way, a p-value less than 0.05 would indicate that the logistic model did not fit the data to a level of statistical significance. For this model, the area under the ROC curve was 0.726. The mean area under the ROC curve for 1,000 bootstrap samples was 0.728 with a 95% confidence interval (CI) of 0.658 to 0.799.

Risk factors were also regressed on the EVAR subsample (n=158), as summarized in Table 2. Statistically significant variables included the use of beta blockers, level of creatinine ≥1.5 mg/dl, history of iliac aneurysm, CABG or PTCA less than 5 years ago, current smoker and ejection fraction less than 30%. Creatinine level was not linearly related to the logit of the outcome, and so was dichotomized as <1.5 vs. ≥1.5 mg/dl.

**Table 2. Predictors of Upper Quartile of Total Cost for EVAR Patients**

| Variable | Comparison | OR | 95% CI Lower | 95% CI Upper | P-value |
|---|---|---|---|---|---|
| Iliac Aneurysm | [unilateral or bilateral] vs. No | 3.30 | 1.34 | 8.14 | 0.009 |
| CABG/PCTA | <5 yrs ago vs. [≥5 yrs ago or none] | 4.94 | 1.67 | 14.65 | 0.004 |
| Ejection Fraction | ≤30 vs. [>30% or not done] | 15.43 | 2.29 | 104.13 | 0.005 |
| Beta Blocker Use | [no or intolerant] vs. Yes* | 3.03 | 1.07 | 8.58 | 0.040 |
| Creatinine ≥1.5 | ≥1.5 vs. <1.5 | 4.61 | 1.48 | 14.35 | 0.008 |
| Current Smoker | Current (within yr) vs. [no or prior (>1 yr)] | 2.47 | 0.96 | 6.33 | 0.060 |

*Yes = Operation day only/preopoeration 1-30 days or chronic >30 days

Note: This model showed adequate fit (p=0.38), with an area under the ROC curve of 0.784. The mean area under the curve from 1,000 bootstrap samples was 0.802 with a 95% CI of 0.712 to 0.883.

EVAR: endovascular repair; CI: confidence interval; CABG: coronary artery bypass graft (surgery); PCTA: percutaneous transluminal coronary angioplasty

In Table 3, significant risk factors (p<0.05) for the EVAR and OPEN repair POCCA models are summarized along with their corresponding cost weightings. These models were "back-tested" on each subset (summarized in Table 4) to determine the difference in predicted versus actual treatment costs, a method which provided strong evidence of ex ante discriminatory ability between OPEN repair and EVAR procedures. Thus, in both subsamples (EVAR vs. OPEN repair) we did not observe a situation where one procedure was dominated by the other. If the predicted cost of an OPEN repair would be less than the cost actually incurred by a patient who had an EVAR procedure, such would constitute evidence of cost savings for physicians choosing the least costly option.

In the final analysis of the costs in Vermont as compared with national estimates (as shown in Table 5), mean EVAR costs were significantly greater than OPEN repair costs, a finding that appears to be unique to the state, or at least to FAHC more specifically. Nationally, this relationship was noticeably reversed, with mean EVAR costs being similar to those observed in our dataset, but totaling significantly less than the nationally reported OPEN repair costs.[8] The National HCUP cost data from 2006 – 2010 showed that mean EVAR costs increased by $5,067 during this time period, while the number of EVAR procedures increased by 4,733. In contrast, national mean OPEN repair procedure costs increased by $11,050, while the number of OPEN procedures decreased by 5,255. The greatest rate of OPEN cost increase was during the most recent years where the number of national EVAR procedures outnumbered OPEN repair procedures by 1.9 to 3.5:1 (between 2006 and 2010).

**Table 3. OPEN and EVAR Predictive Models**

| | Variable | Description | Weighting | P-value |
|---|---|---|---|---|
| OPEN* | Intercept | | -$21,715.63 | 0.105 |
| | Age | In years, continuous | $652.58 | 0.000 |
| | Transfer Status | Not transferred vs. Transferred | $26,075.22 | 0.003 |
| | COPD | No History vs. History | $10,798.38 | 0.002 |
| EVAR** | Intercept | | $35,171.73 | 0.000 |
| | Beta Blocker Use | [No or intolerant] vs. Yes* | -$4,672.12 | 0.001 |
| | Creatinine ≥1.5 | ≥1.5 vs. <1.5 | $3,426.73 | 0.047 |
| | Iliac Aneurysm | [Unilateral or bilateral] vs. No presence | $3,549.79 | 0.001 |
| | Ejection Fraction | ≤30 vs. [>30% or not done] | $4,860.05 | 0.079 |

EVAR: endovascular repair; COPD: chronic obstructive pulmonary disease

*F value 7.76, with a significance level of less than 0.01 (3 and 227 degrees of freedom)
**F value 5.93, with a significance level less than 0.01 (4 and 154 degrees freedom)

**Table 4. Predictive Models on Patient Subsamples**

| | | Patients in Sample (N) | EVAR Predicted Cost Lower (N) | OPEN Predicted Cost Lower (N) |
|---|---|---|---|---|
| OPEN | Lower Half Actual Costs | 115 | 17 | 98 |
| | Upper Half Actual Costs | 115 | 36 | 79 |
| | Total Sample | 230 | 53 | 177 |
| EVAR | Lower Half Actual Costs | 79 | 52 | 27 |
| | Upper Half Actual Costs | 79 | 43 | 36 |
| | Total Sample | 158 | 95 | 63 |

EVAR: endovascular repair

**Table 5. Vermont vs. National HCUP EVAR and OPEN Costs**

| | Mean | SEM | n | p (EVAR vs. OPEN) |
|---|---|---|---|---|
| **Vermont EVAR** | $32,656 | $591 | 159 | 0.0226 |
| **OPEN** | $28,183 | $1,571 | 230 | |
| **National EVAR** | $30,773 | $804 | 33,134 | <0.0001 |
| **OPEN** | $46,019 | $2,096 | 9,561 | |

HCUP: Healthcare Cost and Utilization Project; EVAR: endovascular repair; SEM: standard error of the mean

## 4. Discussion

To date, such risk stratification of patients for either EVAR or OPEN repair has not drawn upon the relative economic costs for either procedure. It has perhaps been generally assumed that the higher device cost of EVAR is offset by the shorter LOS this procedure affords. Similarly, it has perhaps been assumed that the low technical costs for OPEN repair are offset by longer hospital LOS with a requirement for higher intensity care. These assumptions may have used LOS as a surrogate marker for total cost and resource utilization, without a thorough cost analysis. Our study, on the other hand, found a distinct difference between the LOS for EVAR versus OPEN repair. The shorter LOS for EVAR patients did not translate to lower total costs in the center we evaluated. While LOS for OPEN repair more closely correlated with total costs, there was a wide range of total costs for both procedures independent of LOS.

Existing guidelines for treatment of AAA have traditionally been limited to recommendations for radiological planning for intervention and follow-up.[9] They neither routinely extrapolate to individual patients nor provide detailed guidance regarding individualized decision-making for choosing OPEN repair versus EVAR, when such an option exists.[10]

Across any disease, in a state of limited resources, the value of predicting the downstream cost of one treatment versus another is obviously useful to maximizing efficiency. It may also be useful to managing treatments according to patient expectations around alternate future scenarios. Predictive modeling of complex and individual patient circumstances leading to an array of outcomes with corresponding costs has received recent interest.[11] In this study, we utilized a database with the ability to risk-stratify using preoperative clinical data, and examined cost as an outcome in and of itself. To our knowledge, this is the first attempt to apply such tools to the vascular surgery setting, where patients present with a wide range of individual clinical variables and multiple treatment options exist with comparative costs that are not obvious.

While complications were included in the northern New England (NNE) dataset, they were not included in the present analysis. EVAR is well known to be less invasive than "traditional" open AAA repair and clinical reviews have demonstrated lower procedure-related mortality and complication rates.[12-14] However, a successful EVAR is dependent on the dimensions, angulation and quality of the anatomy.[15] EVAR allows patients to be discharged much sooner than those undergoing OPEN repair, and as such leads to reductions in hospital charges for LOS. On the other hand, the technical costs (cf. in particular, stent graft components) of EVAR are responsible for significantly increasing operating room (OR)-related costs. Whereas, it would be logical to presume that such a clinical benefit from a less invasive approach should lead to downstream economic savings, the reality requires further analysis.[16,17]

In the vascular surgery space, it is not surprising that the procedures eluded a thorough cost-prediction analysis, as published studies to date that have focused on the economics of EVAR and OPEN repair have been altogether inconsistent.[3,6] For instance, associated costs of EVAR are sometimes higher and sometimes lower than OPEN repair; in one study, 10-year costs per quality-adjusted life year as well as costs per life year saved were estimated to be higher with EVAR as compared to OPEN repair.[18] Moreover, AAA costing studies to date have either involved single centers or large datasets where average or mean cost of procedures have been the focus, using "averages" of patient costs rather than to focus on the patient's unique set of circumstances that underlie the costs.

A 2007 systematic review observed that all studies to date (based on data between 1995 and 2004) had found that EVAR was more expensive than OPEN repair.[6] To add to this complexity, there has been more recent and alternative evidence to suggest that EVAR is superior in terms of clinical efficacy and quality of life,[19] circumstances which would make it more cost-effective than OPEN repair at least for a targeted subset of patients.[20] Thus, we closely examined a single center dataset to develop a risk-stratified cost methodology that could be personalized to the individual circumstances of patients in our center. Application of the simulation models we propose may add some clarity to this debate, particularly if outcomes and costs can be modeled across a heterogeneous population – although this hypothesis will need to be tested on additional datasets and ultimately, tested prospectively.

The signs in our field would suggest that predictive models are beginning to be embraced by both patients and providers. Patients are increasingly demanding the latest evidence for personalized solutions, and surgeons typically tailor therapeutic measures to the individual patient to achieve an optimal outcome in terms of morbidity and mortality. Considerable efforts have thus been put into the pre-operative identification of high-risk patients for both procedures.[3,21] Perioperative clinical outcomes and complication rates for both of these procedures are established for OPEN repair[9,22,23] and for EVAR,[7,16,17] and as mentioned earlier, were included in the costs up to the point of discharge.

Thus, while a fixed group of patients will be offered only OPEN repair or EVAR exclusively based on guidelines and established clinical experience, the remaining patients will be eligible for either intervention. Unfortunately, absent a prospective trial it was not possible to determine which patients in the dataset would have been eligible for both EVAR and OPEN repair. Equivocality of outcomes need not be in place to evaluate the predictive cost-effectiveness, however. The ratio of incremental cost to incremental benefit achieved would be even more useful in circumstances where the cost, outcomes and ratios between cost and outcomes are complex and unclear. In order to maintain the highest clinical effectiveness, it is envisaged that thresholds could be put in place to firstly predict safety and effectiveness, then, when other factors are predicted to be equal, to predict cost-effectiveness.

Our POCCA models also identified clinical indicators that were predictive of higher cost in EVAR patients, a circumstance that was ultimately driven by the high cost of the requisite device(s). These clinical indicators included cardiac disease, reduced cardiac function, smoking and elevated creatinine. In general, these indicators are indicative of patients with advanced chronic organ system dysfunction. Other studies have found these risk factors associated with longer rates of stay and higher complication rates. Interestingly, in the EVAR model, use of beta-blockers was associated with lower costs of care, a finding we are presently investigating.

In the case of OPEN repair, we identified four factors (age, COPD, transfer and history of bypass) as being significant associated with high cost OPEN repairs. Age and COPD have, in other studies, been identified as independent predictors of poor outcomes.[3,23] Patients transferred from outside facilities are more likely to be "sicker" with active comorbidities, clinical instability and possibly under resuscitation. "History of bypass" as a significant risk factor is not surprising, as this is a marker for significant atherosclerotic obstructive disease and aneurysmal disease.

As has been suggested by Tarride, *et al.*,[20] risk-stratification may make EVAR a more cost-effective option for certain subsets of patients, or for certain clinics and settings that are similar to those found in Vermont. On the other hand, our forward simulation approach would estimate when OPEN repair is predicted to be the most cost-efficient.

Optimizing quality involves considering cost and resource allocations along with clinical outcomes particularly in situations where different therapeutic options are available. Our analysis involved patients treated at a single center. Multiple factors contribute to the total costs of a specific intervention and are often unique for a given institution. These may include:

- An institution's patient base (primary facility or referral center);
- Facilities available (Fixed imaging in OR, intensive care unit (ICU) availability, OR technical capabilities and support;
- Excellence of medical personnel (surgeons and OR/ward staffs);
- Contracting capabilities of the administrative branch of that same hospital (device cost); and
- Local definitions of cost (direct, indirect).

Predictive modeling at a given hospital will change with time – indeed, as the above factors change over time. Strategies for treating patients with AAA at a given hospital should be developed from national standards and from clinical outcomes and cost data unique to that hospital. For instance, a lack of ICU beds may be favorable to increase the relative proportion of EVAR cases. Conversely, construction of a new ICU may lead to better or more efficient care with OPEN repair.

Because of local variances in facilities, personnel, and cost definitions, it is likely that optimizing cost and quality involves different care formulas at different hospitals. While some of the clinical preoperative factors identified here have broad applicability to predicting high hospital cost of groups of AAA patients, the same patient may be optimally treated using different methods at different hospitals depending on local expertise, facilities and contracting.

The relative hospital costs for EVAR and OPEN repair procedures in Vermont as compared to national data certainly deserve further discussion. Historically, data between 1995 and 2004 showed that EVAR was consistently more expensive than OPEN repair surgery.[6] Our analysis of HCUP data between 2006 and 2010 shows the opposite relationship; annual national mean OPEN repair costs were always greater than EVAR costs during that time period (HCUP cost data for EVAR and OPEN repair procedures were not available before 2006). However, nationally the number of EVAR procedures has grown sizably in recent years, while the number of OPEN repair procedures has, as a result, declined. As of 2010, the number of EVAR procedures outnumbered OPEN repair procedures by 3.5:1. In sharp contrast, mean OPEN repair costs at FAHC are significantly lower than EVAR costs, while the number of OPEN repair procedures outnumbers EVAR by 1.4:1. These findings suggest that there is an inverse relationship between hospital surgical procedure experience and associated costs that should also be taken into account when predicting and making any policy decisions based on the cost of EVAR versus OPEN repair of AAA.

This study is not without certain limitations; foremost of which is that risk factors for EVAR and OPEN procedures were not mutually-exclusive in the present study. In other words, patients could theoretically have been predicted to be in the upper (or lower; data not shown) quartile of cost for both EVAR and OPEN repair. However, the coefficient estimates and statistical significance of risk factors for the predictive models were estimated based on subsamples that were, indeed, mutually exclusive. EVAR risk factors weightings were estimated from data on actual EVAR patients and OPEN risk factors, from actual OPEN repair patients. Clearly, also, the ratio of EVAR to OPEN repair treatments was far from unity and this flies in the face of the assumption that all patients who underwent EVAR could have equivocally undergone OPEN repair, or vice versa.

## 5. Conclusions

Our results suggest EVAR versus OPEN repair decisions can be optimized to individual patient circumstances. As such, we anticipate that care pathways can eventually be tailored to the unique needs of AAA patients, given the option of undergoing either approach, with counterfactual scenarios tailored to the patient at the point-of-care.

Vermont passed monumental legislation in October 2011 to tackle enduring problems in its health economy—an increasingly high cost of care and health expenditure, a continually uninsured population and variation in terms of access to care. While these challenges were not unique to Vermont, the state's size, socially liberal political climate and history of health reforms aimed towards a single-payer system, has allowed for more momentum towards improving outcomes and reducing costs than can be found in any other U.S. state. We believe that as these reforms move forward and expand across other settings, POCCA will become central to improving efficiency at the point of care.

Around the world, health economic analyses are increasingly being used to influence healthcare reimbursement decisions and to develop cost-conscious guidelines.[24] Most of such analyses are based on patients who participate in highly controlled clinical trials. While these guidelines occasionally simulate the future effects of policy changes on a hospital or even national budget, based on the mean effect sizes, they have yet to parlay this type of simulation to the individual patient level, particularly where such patients may experience individual or exceptional circumstances rather than the circumstances calculated to befall the mean. The usefulness of our POCCA method to estimate future costs in heterogeneous populations hinges on a) non-obvious choices of treatments being available, b) weighting of individual patient needs and desires, c) the expertise and preferences of the treating physician(s), and d) the payer's willingness and/or ability to pay for a cost of treatment that varies with intensity of treatment. When resources are constrained, clinically-based patient outcomes may no longer be the sole measures for risk stratifying specific procedures. Quality assessment must also include resource utilization and costs in the overall comparison of treatment options. Simulating cost data with clinical data sets allows for the development of modeling tools capable of risk-stratifying patients ahead of their surgery. It is envisioned that the models reported herein could be uniquely modified for any medical center based on regionally specific combinations of clinical expertise, patient-engagement, contracting, and resource allocation.

Cost-prediction modeling, we contend, will spearhead the development of disease- and population-specific algorithms that can illustrate future costs in real-time, as scenarios are compared and decisions made, so that a unique patient receives the highest efficiency of care, at the right time and in the right setting. Our methodology may be applied across any array of procedures for which multiple pathways exist and corresponding, personalized data are available.

## Declaration of Competing Interests

The authors declare they have no competing interests.
